# No indication of highly pathogenic avian influenza infections in Dutch cows

**DOI:** 10.3168/jdsc.2024-0703

**Published:** 2025-03-12

**Authors:** N.D. Fabri, I.M.G.A. Santman-Berends, C.A.J. Roos, G. van Schaik, J. het Lam, E.A. Germeraad, M.H. Mars

**Affiliations:** 1 Royal GD, 7418 EZ Deventer, the Netherlands; 2 Faculty of Veterinary Medicine, Department of Population Health Sciences, Utrecht University, 3584 CM Utrecht, the Netherlands; 3 Wageningen Bioveterinary Research, 8221 RA Lelystad, the Netherlands

## Abstract

In March 2024, an infection with highly pathogenic avian influenza (HPAI) virus subtype H5N1 of the HA clade 2.3.4.4b was detected in dairy cows in Texas. Following this first detection, the virus was identified in multiple states in the United States, with spillover into other mammals. This raised concerns in the Netherlands that despite having an effective monitoring and surveillance system, H5N1 virus infections in Dutch cattle could have occurred. The aim of this study was therefore to retrospectively screen Dutch cattle for HPAI virus infections for the period between 2022 and 2024. A total of 2,190 archived cattle serum samples from 367 different herds were selected, with an average of 6 samples per herd. These samples were tested using an influenza A blocking ELISA. A Luminex H5/H7 assay was used to confirm and subtype ELISA-positive samples. The influenza A blocking ELISA detects antibodies against all influenza A virus subtypes, whereas the Luminex assay can differentiate antibodies against the surface proteins haemagglutinin (HA or H) 5 and 7 (H5/H7), and neuraminidases (NA or N) 1 to 9 (N1–N9). Four samples (0.2%) reacted positive in the influenza A blocking ELISA, all from different herds located in different parts of the Netherlands. However, all 4 samples tested negative in the Luminex H5/H7 assay, indicating that the samples did not have antibodies against H5, H7, and N1 to N9. The 99.8% specificity of the ELISA may explain the ELISA-positive results. The ELISA used in this study is a multispecies test that has not yet been validated for use in cattle. The duration of detectable antibodies in cattle is unknown, as is the sensitivity to detect past infections with influenza A virus subtypes in cattle for the Dutch situation. Therefore results should be interpreted with caution. The lack of detected antibody responses in cattle in the Netherlands from 2022 to 2024 aligns with the absence of any indication for clinical cases in Dutch cattle over recent years in the national monitoring and surveillance system.

## Highlights

• Concerns were raised about HPAI virus infections in Dutch cattle.
• Serum samples from cattle collected between 2022 and 2024 were tested for HPAI virus.
• Four samples tested (false) positive in an influenza A blocking ELISA.
• No antibodies were detected in the confirmatory Luminex H5/H7 assay.
• There is no indication of HPAI virus infection in Dutch cattle.

## Graphical Abstract

**Figure:**
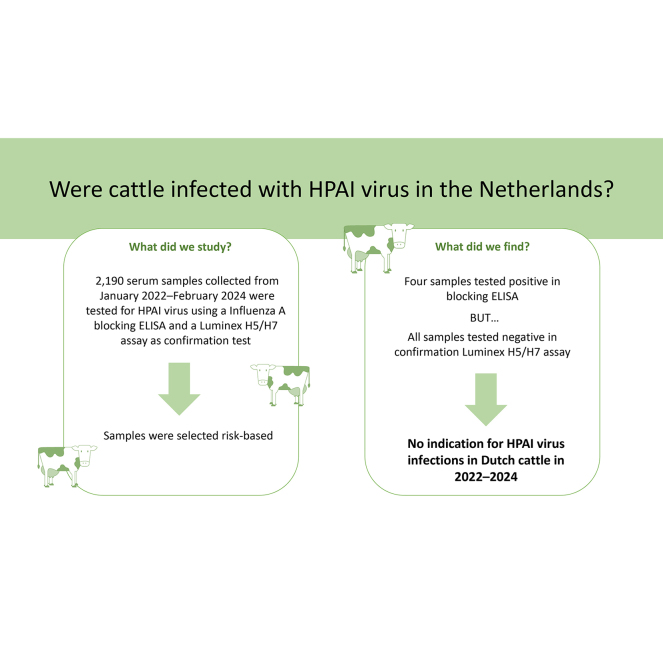
**Summary:** After highly pathogenic avian influenza (HPAI) virus was detected in dairy cows in the United States, concerns were that infections could have occurred in Dutch cattle despite having an effective monitoring and surveillance system. A total of 2,190 archived cattle serum samples from 367 different herds collected between January 2022 and February 2024 were selected risk-based for testing. Although 4 samples initially tested positive in an influenza A blocking enzyme-linked immunosorbent assay (ELISA), a confirmatory Luminex H5/H7 assay showed that none of the samples had antibodies against H5, H7, and N1 to N9. The positive results in the ELISA were likely false positive. In conclusion, there is no indication that HPAI virus subtype H5N1 has circulated in cattle in the Netherlands between 2022 and 2024. This aligns with the absence of any indication for clinical cases in Dutch cattle over recent years in the national monitoring and surveillance system.

Avian influenza is a disease caused by influenza A virus, belonging to the virus family *Orthomyxoviridae*. The different subtypes of this virus can be divided into low pathogenic and highly pathogenic, based on lethality in chickens (WOAH, 2024). Highly pathogenic avian influenza (**HPAI**) virus specifically poses a threat to both wild birds and poultry globally. In particular, the subtype H5N1, which is persisting in wild bird reservoirs, is of great concern because of its spillover into mammals, including humans. H5N1 virus infections have been reported in multiple animal species, including seals, sea lions, foxes, polecats, martens, minks, and domestic cats (e.g., Klopfleisch et al., 2007; Thiry et al., 2007; Marschall and Hartmann, 2008; Floyd et al., 2021; Rijks et al., 2021; Agüero et al., 2023; Bordes et al., 2023; Chestakova et al., 2023; Elsmo et al., 2023; Mirolo et al., 2023; Sillman et al., 2023; Ulloa et al., 2023; EFSA et al., 2024). Until recently, cattle have not been affected with HPAI virus subtype H5N1, but infections have been found with other subtypes of the influenza A virus, where they act as dead-end hosts (Sreenivasan et al., 2019). Cattle are, however, a natural reservoir of influenza D virus (Ruiz et al., 2022).

In March 2024, HPAI virus subtype H5N1 of the HA clade 2.3.4.4b was detected in dairy cows in Texas (Burrough et al., 2024; Caserta et al., 2024; Oguzie et al., 2024). Clinical signs included decreased feed intake and rumination time, abrupt drop in milk production, lethargy, mild respiratory signs, moderate fever, abnormal bowel movements, and colostrum-like yellow to brown-red milk with an abnormal consistency (Caserta et al., 2024; Oguzie et al., 2024). After the first detection in Texas, this virus was identified in dairy cattle herds across multiple states in the United States, with spillover to other mammals, including humans (Burrough et al., 2024; Caserta et al., 2024; USDA, 2024; Uyeki et al., 2024). Additionally, it has been shown that the virus can persist on milking unit surfaces (Le Sage et al., 2024), and there are strong indications of cow-to-cow transmission of the virus through contaminated milk and milking equipment (Burrough et al., 2024; Caserta et al., 2024; Halwe et al., 2025). Furthermore, animal transport has been suggested as a mode of spread (Burrough et al., 2024).

The situation regarding HPAI virus subtype H5N1 in the United States raised concerns in the Netherlands. The Dutch cattle health surveillance system (**CHSS**) is effective for detection of disease (Santman-Berends et al., 2016; Veldhuis et al., 2016; Vredenberg et al., 2022). Therefore, expectations would be that no large, clinical HPAI virus outbreak in cattle could go undetected. However, it could be possible that sporadic H5N1 virus infections in Dutch cattle might have occurred because of exposure due to the multiple outbreaks of HPAI virus in wild birds, poultry, domestic cats and wild carnivores in the Netherlands between 2021 and 2023 (Bordes et al., 2023; Chestakova et al., 2023; Wolters et al., 2024). At Royal GD (Deventer, the Netherlands), many serum samples of Dutch cattle are tested for various endemic pathogens as part of routine diagnostics in cattle herds. From these samples, every week a random block with 96 serum samples was collected and stored at −20°C to enable the possibility for retrospective screening. This set of samples provided the opportunity to retrospectively screen Dutch cattle for HPAI virus infections. The aim of this study was to evaluate whether or not cattle in the Netherlands were infected with HPAI virus.

An influenza A blocking ELISA from Idexx (Westbrook, ME) has been used previously in the Netherlands to detect influenza A antibodies in serum from poultry, wild birds, horses, minks, and pigs. In poultry, the sensitivity is estimated at 89% to 100% (data Royal GD) and the specificity at 99.8%. This ELISA is designed to detect antibodies against the nucleoprotein (**NP**) of influenza A viruses in multiple species and has been validated for several species, but not yet for cattle (L. Soderlund, Idexx, personal communication). Before starting our study, sensitivity in cattle was unknown, but now the performance of this ELISA in acute outbreaks in dairy cattle in the United States has been described to be good (Giménez-Lirola et al., 2024). This blocking ELISA was also used by the German reference institute Friedrich-Loeffler-Institut (Greifswald, Germany) in a H5N1 challenge in cattle (Kalthoff et al., 2008). Before our study, 10 sera of influenza D–infected cattle with high influenza D HI (hemagglutination inhibition) titers were tested in the influenza A blocking ELISA at Royal GD in order to check for cross-reactions with antibodies against the bovine influenza D virus. All had negative results (data Royal GD).

From January 2022 to February 2024, a total of 11,200 frozen cattle serum samples were available. These were anonymized for further analysis. To enable detection of 1% prevalence with 99% confidence using the 90% sensitivity of the influenza A blocking ELISA in poultry was used and a 100% specificity (including the confirmation), 512 samples of serum samples should be tested (Sergeant, 2018). Given that the sensitivity of the test is based on poultry samples and the test characteristics in cattle samples at the start of the project were unknown, it was decided to test at least the double amount of samples to ensure sufficient sensitivity. Testing more samples would also have enabled us to correct for repeated measures within herds, as the samples within a serum block were not independent from one another because often samples from multiple cattle in a herd are submitted and end up in the same block.

Samples were classified as high-risk samples based on the following criteria:

(1) The cow from which the sample was taken had to be born in the Netherlands, as cows born in other countries would have an unknown risk of getting infected with HPAI virus. Furthermore, by including only cattle born in the Netherlands, the results would reflect the conditions within the country.
(2) The cow from which the sample was taken needed to be at least 2 yr old at the moment of sampling, so it was likely to have been on pasture during its life. Also, data from the United States suggested that clinical signs were more commonly reported in older cows (Burrough et al., 2024).
(3) The cow needed to be housed in a 2-digit postal code area where HPAI virus had been detected during our study period in either wild birds, cats, or poultry (Figure 1), based on data from the Faculty of Veterinary Medicine at Utrecht University (Utrecht, the Netherlands), the Netherlands Food and Consumer Product Safety Authority (Utrecht, the Netherlands), and Wageningen Bioveterinary Research (**WBVR**; Lelystad, the Netherlands).

**Figure 1:**
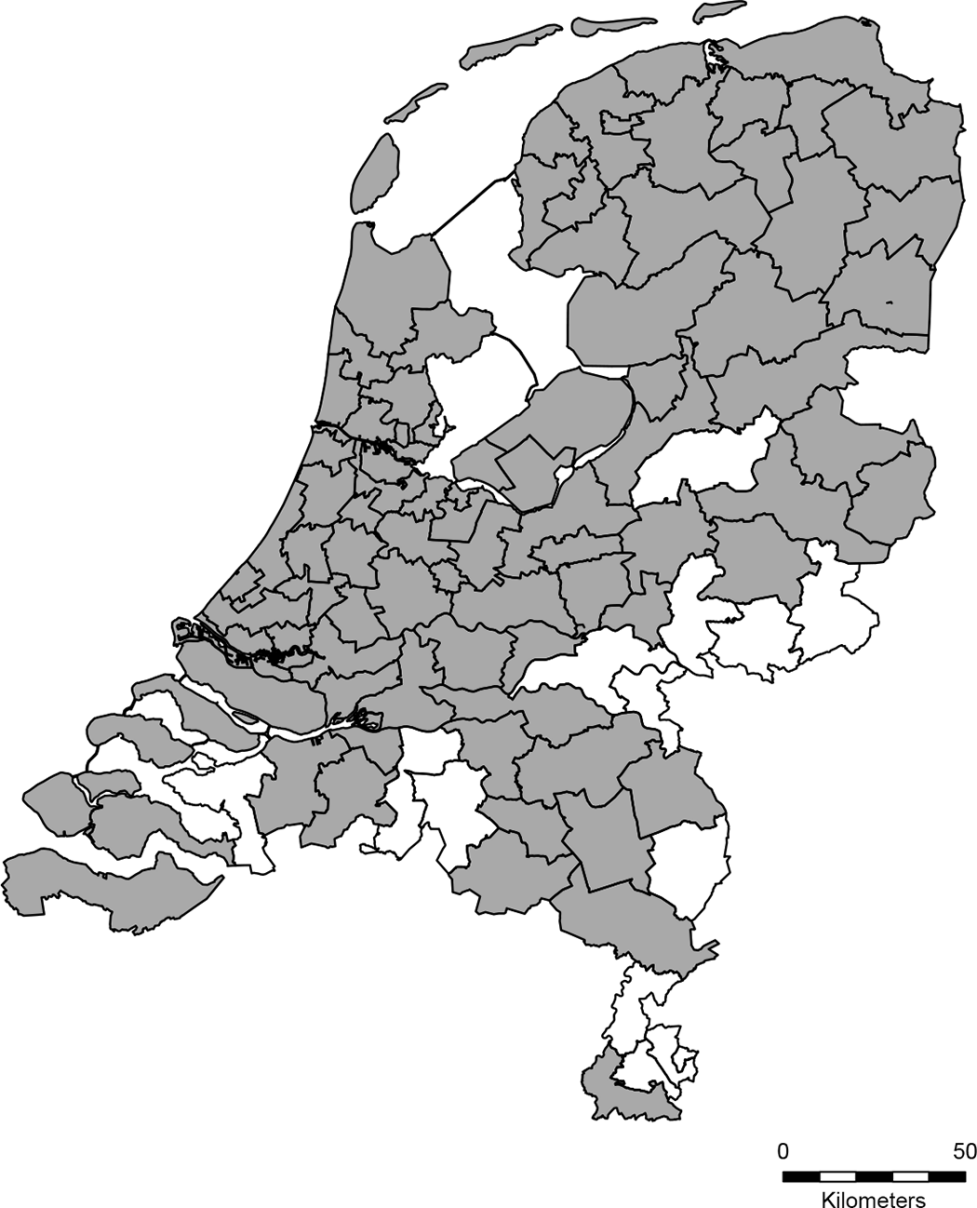
Map showing the 2-digit postal code areas in the Netherlands where HPAI virus was found in wild birds, domestic cats, or poultry during the period between January 2022 and February 2024 (gray shaded areas).

Samples from cattle that did not match these criteria or for which data were missing were not classified as high-risk samples.

Of the 11,200 stored serum samples, 2,513 (22%) were classified as high-risk samples. Selecting individual high-risk serum samples from each block was logistically challenging and prone to selection errors. Therefore, it was decided to select complete serum blocks to be included in the study if at least 35% of the serum samples in that serum block were classified as high-risk samples. This resulted in 23 serum blocks with 2,206 serum samples, of which 1,062 (48%) were high-risk samples. Of these 2,206 samples, 768 were collected in 2022 (of which 376 were high-risk samples), 1,055 in 2023 (520 were high-risk samples), and 383 in January and February of 2024 (166 were high-risk samples).

Of all 2,206 serum samples 2,190 were tested individually with the Idexx influenza A blocking ELISA according to the manufacturer's protocol. The remaining 16 samples could not be tested because the volume of serum was too small. Samples were deemed to be positive with a sample to negative ratio (**S/N ratio**) below 0.5. The influenza A blocking ELISA does not differentiate between the different subtypes of the influenza A virus. The ELISA-positive serum samples (accompanied by the same number of ELISA-negative serum samples) were sent to WBVR for confirmation.

The Luminex H5/H7 assay, a multiplex assay based on Luminex technology and previously described (Germeraad et al., 2019), was used for confirmation and differentiation of ELISA-positive samples. The assay contained subsets of previously described recombinant proteins haemagglutinin and neuraminidase: H5 (n = 5), H7 (n = 3), N1 (n = 3), and N2 to N9 (n = 1 for each), and one NP as positive control. The recombinant proteins were coupled to color-coded magnetic beads. Binding of serum antibodies to the proteins on the beads was detected by fluorescent secondary antibodies, and using the Luminex MAGPIX device, the different beads were identified. This allowed subtyping of antibodies of HA 5 and 7, and NA 1 to 9. Through the strategy of combining the influenza A blocking ELISA and test positive samples in the Luminex, the combined specificity of the tests is assumed to be 100%.

The 2,190 tested serum samples originated from cattle from 367 different herds with an average of 6 samples per herd (median: 3). The interquartile range of the number of samples per herd was 1 to 5. In total 1,051 of the tested serum samples were classified as high-risk. These high-risk samples originated from cattle in 142 different herds with an average of 7 samples per herd (median: 2). The other 1,139 serum samples were classified as low-risk based on age (<2 yr) or geographic location or missing data. The herds from which the samples originated were distributed across the Netherlands, however, not all 2-digit postal code areas were represented (Figure 2A). This was the case for all the tested serum samples as well as the tested high-risk serum samples (Figure 2B). The number of serum samples tested per 2-digit postal code area was in line with the cattle density in the Netherlands, and the 2-digit postal code areas that were not represented were mainly areas with a low cattle density (Figure 2C).

**Figure 2:**
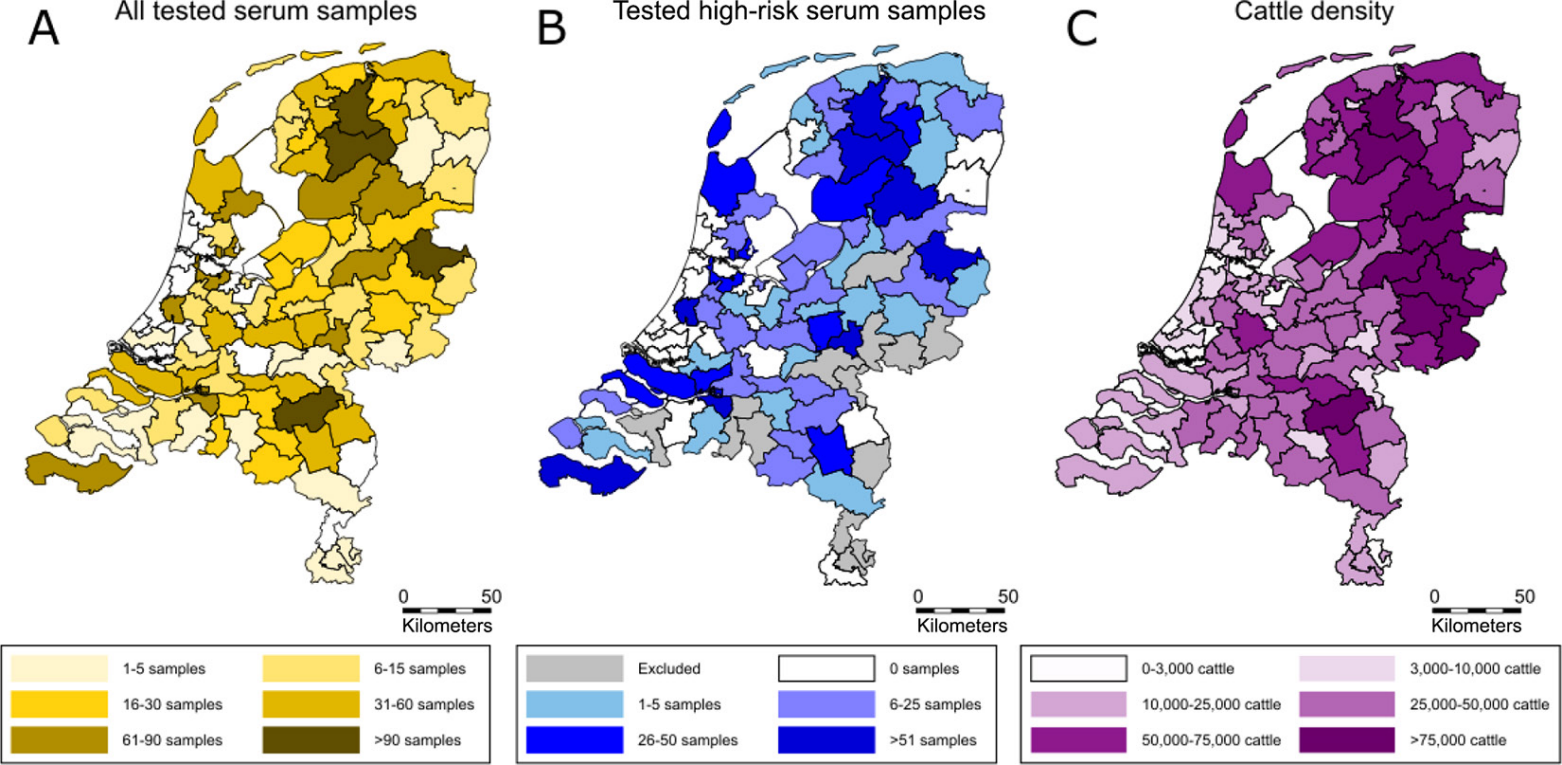
Number of tested serum samples (A; n = 2,190) and number of tested serum samples that were classified as high-risk samples (B; n = 1,051), alongside the cattle density in the Netherlands (C) per 2-digit postal code area.

Of the 2,190 tested serum samples, 4 samples (0.2%) reacted in the influenza A blocking ELISA (Table 1), all from different serum blocks. Of the 4 seropositive serum samples, 3 were classified as high-risk samples, and one serum sample was not. The 4 samples were submitted in 2022 (n = 3) and 2023 (n = 1) and were collected from cattle from 4 different herds located in different areas in the Netherlands. Subsequently, these 4 ELISA-positive serum samples, together with 4 random ELISA-negative serum samples, were send to WBVR for confirmation. In the Luminex H5/H7 assay, all 8 samples turned out to be negative (Table 1). This means that no antibodies against H5/H7 or NA 1 to 9 were detected. In one of the samples, some reaction against NP was seen (below cut-off). This indicates that the 4 initial positive samples might have been false positives in the influenza A blocking ELISA, which fits with the 99.8% specificity as described in poultry samples (data Royal GD). The average S/N ratio of serum samples that were negative in the influenza A blocking ELISA was 1.014 (median 1.013) with an interquartile range of 0.940 to 1.095.

**Table 1:** Results of the influenza A blocking ELISA (performed at Royal GD) and Luminex H5/H7 assay of the 8 samples sent for confirmation to Wageningen Bioveterinary Research^1^

Sample number	Influenza A blocking ELISA		Luminex assay
	S/N ratio	Result	
1	**0.265**	Positive	Negative
2	**0.402**	Positive	Negative
3	**0.485**	Positive	Negative
4	**0.476**	Positive	Negative
5	1.114	Negative	Negative
6	1.135	Negative	Negative
7	1.052	Negative	Negative
8	1.090	Negative	Negative

1 Positive results (S/N ratio ≤0.5) are indicated in bold.

The results in this study are in line with the general census that even though the HPAI virus subtype H5N1 has infected herds in multiple states in the United States (Burrough et al., 2024; Caserta et al., 2024), there is no indication of any spillover events in European countries at the moment. A study in Germany showed no positive results after testing 1,400 bovine serum samples for HPAI antibodies and 350 bulk milk samples for the virus (FLI, 2024). Furthermore, no HPAI virus infections have been found in Italian cattle according to the Italian Health Authority and Research Organization (IZSVe, 2024). Furthermore, the HPAI virus found in cattle in the United States belongs to clade 2.3.4.4b genotype B3.13, a genotype that has not been detected in Europe so far (EFSA et al., 2024).

The influenza A blocking ELISA used in our study has not been validated for use in cattle samples by Royal GD because appropriate samples for such a validation study were not available. To date, no validation studies of serological tests for detection of antibodies to influenza in cattle have been published. However, by design, blocking ELISA are generally suitable for detecting antibodies across different species. Moreover, the same ELISA has been used successfully in cattle in other influenza studies (e.g., Kalthoff et al., 2008; Giménez-Lirola et al., 2024), although these studies only included animals shortly after infection.

The tested serum samples were not a fully random selection of the Dutch cattle population because the selection was a convenience sample from submissions for disease control programs or clinical cases from veterinarians and farmers to the diagnostic laboratory of Royal GD. Therefore, Dutch veterinarians and farmers that never submit serum samples to Royal GD are not included. However, we believe that the samples were random relative to the outcome variable because all herds have the same risk of getting infected, except for their location in the Netherlands. Furthermore, samples were clustered because often multiple samples from one herd were stored in a serum block. Cattle from the same herd are more likely exposed to the same risk factors, or could have infected one another. As described in previously, we have tested more than double the number of samples that were strictly necessary in order to compensate for this clustering and for the unknown sensitivity of the influenza A blocking ELISA. Although not all 2-digit postal code areas where HPAI virus was found in wild birds, cats, and poultry during our study period were represented, the geographic distribution in our sample reflects the cattle density in the Netherlands. At animal-level and assuming a test sensitivity of 90%, a sample of 512 seronegative cattle would allow for the conclusion that seroprevalence would be below 1% with 99% confidence. The same conclusion could be drawn with a sample of 919 seronegative cattle if sensitivity of the influenza A blocking ELISA would be as low as 50%. In our study, 1,051 high-risk cattle tested negative. In addition to the unknown sensitivity, duration of antibody response is unknown, therefore results should be interpreted with caution.

By selecting the serum samples using a risk based method, we maximized the probability of detecting HPAI virus in cattle, if present. The method used in this study implied multiple criteria to classify samples as high risk, such as being older than 2 yr at the time of sampling. This criterion was included based on the assumption that cattle older than 2 yr were likely to have grazed at some point in their lives and could have come into contact with wild bird droppings and carcasses. In the Netherlands, almost 75% of all cattle >2 yr of age are field-grazed, whereas calves are more often kept indoors (CBS, 2022). In addition, older cows are more likely to have been in contact with the virus during their life. Furthermore, cattle older than 2 yr are likely to be lactating, unlike younger cattle, and if an important mode for cow-to-cow transmission involves contaminated milk and milking equipment, lactating cows are more likely to become infected (Le Sage et al., 2024; Halwe et al., 2025). Burrough et al. (2024) claimed that in the United States, the infection has been found more commonly in older, multiparous, cows.

In the Netherlands, a CHSS is in place that combines passive reporting, diagnostic and postmortem examinations, random surveys for prevalence estimation of endemic diseases, and data analysis (Santman-Berends et al., 2016, Veldhuis et al., 2016; Vredenberg et al., 2022). The aim of the CHSS is to detect (emerging) diseases and monitor trends and developments in cattle health. In past years, no evidence of clinical cases consistent with an HPAI virus infection has been found within the CHSS, and therefore it was hypothesized that HPAI virus has not circulated among cattle in the Netherlands. The results of our study support this hypothesis. The national CHSS is key for early detection of any possible future outbreaks of HPAI infections in cattle and is well imbedded in the zoonoses structure in the Netherlands (Van der Giessen et al., 2022).
